# Transcription profiling of a recently colonised pyrethroid resistant *Anopheles gambiae *strain from Ghana

**DOI:** 10.1186/1471-2164-8-36

**Published:** 2007-01-29

**Authors:** Pie Müller, Martin J Donnelly, Hilary Ranson

**Affiliations:** 1 Vector Group, Liverpool School of Tropical Medicine, Liverpool, L3 5QA, UK

## Abstract

**Background:**

Mosquito resistance to the pyrethroid insecticides used to treat bednets threatens the sustainability of malaria control in sub-Saharan Africa. While the impact of target site insensitivity alleles is being widely discussed the implications of insecticide detoxification – though equally important – remains elusive. The successful development of new tools for malaria intervention and management requires a comprehensive understanding of insecticide resistance, including metabolic resistance mechanisms. Although three enzyme families (cytochrome P450s, glutathione S-transferases and carboxylesterases) have been widely associated with insecticide detoxification the role of individual enzymes is largely unknown.

**Results:**

Here, constitutive expression patterns of genes putatively involved in conferring pyrethroid resistance was investigated in a recently colonised pyrethroid resistant *Anopheles gambiae *strain from Odumasy, Southern Ghana. RNA from the resistant strain and a standard laboratory susceptible strain, of both sexes was extracted, reverse transcribed and labelled with either Cy3- or Cy5-dye. Labelled cDNA was co-hybridised to the *detox chip*, a custom-made microarray containing over 230 *A. gambiae *gene fragments predominantly from enzyme families associated with insecticide resistance. After hybridisation, Cy3- and Cy5-signal intensities were measured and compared gene by gene. In both females and males of the resistant strain the cytochrome P450s *CYP6Z2 *and *CYP6M2 *are highly over-expressed along with a member of the *superoxide dismutase *(*SOD*) gene family.

**Conclusion:**

These genes differ from those found up-regulated in East African strains of pyrethroid resistant *A. gambiae *and constitute a novel set of candidate genes implicated in insecticide detoxification. These data suggest that metabolic resistance may have multiple origins in *A. gambiae*, which has strong implications for the management of resistance.

## Background

Pyrethroid-treated bednets are the mainstay of malaria control programs in sub-Saharan Africa [1]. Pyrethroids are a class of fast acting and highly effective insecticides showing low mammalian toxicity levels. Currently no other insecticide class with similar efficacy and specificity is available for use on bednets. Therefore the emergence of pyrethroid resistance threatens to compromise the successful use of insecticide-treated materials [2-6].

Two mechanisms of insecticide resistance play an important role in mosquitoes, target site insensitivity and metabolic resistance [7]. Target site insensitivity to pyrethroids in *Anopheles gambiae *is associated with a single point mutation, commonly referred to as knock-down resistance (*kdr*). The *kdr *allele leads to a modification of the voltage-gated sodium channel protein resulting in reduced sensitivity to both DDT and pyrethroids [8,9]. Metabolic resistance is generally associated with three enzyme families: cytochrome P450 monooxygenases (P450s), carboxylesterases (COEs), and glutathione-S-transferases (GSTs) [10]. In *A. gambiae *a total of 111 putative P450s, 51 COEs, and 31 GSTs genes have been identified [10]. Though the groups contain large numbers of genes, these enzyme families have multiple roles in the insect and only a small number are thought to be directly involved in insecticide metabolism. Enzymes from all three families have been implicated in conferring resistance to pyrethroids in other insect species (e.g. [11-15]). In *A. gambiae *increased levels have been observed for the *GSTE2 *transcript in the permethrin tolerant RSP strain from Western Kenya [16]. Increased expression of *GSTE2 *was also observed in the ZAN/U strain, from Zanzibar, resistant to DDT [17,18]. *GSTE2 *transforms DDT into a non-toxic product by dehydrochlorination [17]. However, its role in detoxifying pyrethroids requires further investigations. In addition to elevated expression of *GSTE2*, over-expression of two P450 genes, *CYP6Z1 *and *CYP325A3*, was observed in adult mosquitoes of the RSP strain [16,19].

Current data on gene expression levels related to metabolic resistance in *A. gambiae *is limited to strains from East Africa that were colonised over a decade ago. Despite the high levels of pyrethroid resistance found in malaria vectors in West Africa [2,3] there have been no previous studies on gene expression patterns associated with metabolic resistance mechanisms operating in this region.

Noteworthy, studies on *kdr *revealed different alleles to be predominant in East and West African field populations suggesting that alternative mutations may evolve in response to insecticide exposure [8,9]. Given the differences in selection pressures due to variations in the use and type of insecticide, and possible restrictions to gene flow across the African continent [20], we would predict similar differences in metabolic resistance mechanisms.

The presence of multiple resistance mechanisms within *A. gambiae *would have considerable implications for the success of vector intervention and the monitoring of ongoing control programs, demanding the development of appropriate tools to monitor resistance in field populations. The development of such tools requires the identification of candidate genes, and with this goal in mind, we used the *detox chip*, a custom-made DNA microarray chip, developed in our laboratories [16], to measure expression patterns in a recently colonised pyrethroid resistant strain from Southern Ghana.

## Results

### Gene expression associated with resistance status

The expression levels of genes putatively involved in insecticide resistance were compared by co-hybridising labelled RNA from the pyrethroid resistant Odumasy strain and the standard susceptible Kisumu strain to a custom made microarray [16]. As both sexes of the resistant Odumasy strain showed high resistance to permethrin hybridisations were performed for both females and males. Percentage survival after a one hour exposure to 0.75% permethrin, although statistically significant (χ^2^-test, *p *= 0.0045), differed only slightly between sexes with 74% (*N *= 297) for females and 62% (*N *= 224) for males. Out of 233 gene probes on the microarray, 40 genes were significantly (*p *< 0.001) differentially expressed among females, and 50 genes among males, between the two strains (see Additional file 1). Among these genes 14 are common to both sexes and showed the same expression patterns (correlation: *r = *0.91, *p *< 0.001). Of particular interest are genes that show increased transcript levels in both sexes of the resistant strain. We set a cut-off value of two-fold differential expression to select biologically relevant candidate genes among those showing a significant difference in transcript levels, and focus on genes that are over-expressed in both sexes of the resistant strain.

**Figure 1:**
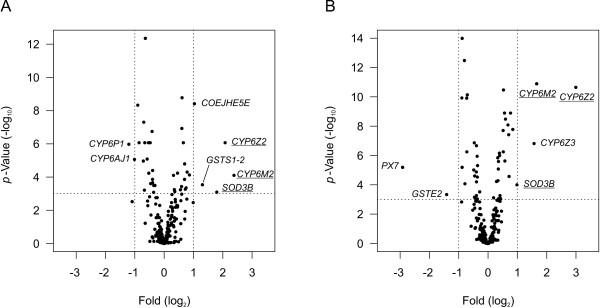
**Microarray analysis comparing the permethrin resistant Odumasy strain with the standard susceptible Kisumu strain**. Data are shown for the differences in expression levels between the permethrin resistant Odumasy strain and the standard susceptible Kisumu strain for both females (A) and males (B). Each dot represents the mean estimates, fold (*M*) and *p*-value (adjusted for multiple testing), for one gene from the complete microarray experiment. Names are shown for genes that are at least two-fold differentially expressed. Underlined names show genes that are common to both the female-female and male-male comparison. Horizontal line represents the level of significance α = 0.001, and vertical lines indicate two-fold change threshold.

In total, ten genes showed a greater than two-fold difference in expression between the strains (Figure 1 and Table 1). Six of these genes were expressed at higher levels in the Odumasy strain and four showed higher expression in the susceptible strain. The genes over-expressed in the resistant strain include three cytochrome P450s (*CYP6M2*, *CYP6Z2 *and *CYP6Z3*), a sigma class GST (*GSTS1-2*), a carboxylesterase, *COEJHE5E*, and a superoxide dismutase (*SOD3B*). *GSTS1-2 *was over-expressed in females while *CYP6Z3 *over-expression was detected in males only. In both males and females, the most pronounced differences were in the expression of two *CYP6 *P450s, *CYP6M2 *and *CYP6Z2*. *CYP6Z3 *was significantly over-expressed (3.0-fold) in males but the data for the female comparison was not significant. This may reflect the very low expression levels of this gene in adult mosquitoes rather than any sex specific differences in expression (*CYP6Z3 *expression was undetectable in adults via quantitative RT-PCR [19]). Indeed the female-male comparisons did not detect any differential expression of this gene (see Additional file 1). All three P450s over-expressed in the resistant strain are clustered in a group of 14 *CYP6 *genes on chromosome arm 3R [19].

Two of the transcripts over-expressed in the resistant strain are derived from genes with alternative splicing. Two transcripts have been detected from the superoxide dismutase gene, *SOD3 *(Ranson, unpublished data), one of which (*SOD3B*) shows elevated expression in both males and females from the resistant strain. Both *SOD3B *and the alternative transcript *SOD3A *share a common N terminal but differ in their C termini. *SOD3A *transcripts are detectable in both male and female *A. gambiae *although in the Kisumu strain, the *SOD3B *transcript is consistently found at higher levels (Pignatelli and Ranson, unpublished data). The sigma class GST, *GSTS1*, also contains a common 5' exon that can be spliced to alternative 3' exons generating two distinct transcripts, *GSTS1-1 *and *GSTS1-2 *[21]. As for *SOD3 *both transcripts are detectable by quantitative RT-PCR in adults from both sexes [22]. The data from this study shows elevated expression of both transcripts in females from the resistant strain versus females from the susceptible strain (see Table 1 and Additional file 1) but no significant difference between the males.

Four genes were expressed at higher levels in the susceptible versus the resistant strain (Figure 1 and Table 1). Surprisingly these include *GSTE2*, the gene associated with both pyrethroid and DDT resistance in *A. gambiae *from East Africa [16-18]. One P450, *CYP6P1 *is a member of a cluster of nine P450 genes on chromosome arm 2R [23] while the second, *CYP6AJ1 *(previously named *CYP6AK1*), is located on chromosome arm 3L and has also been found under-expressed in the DDT resistant ZAN/U strain [16]. The fourth gene, *PX7*, is a peroxidase. As for *CYP6Z3*, *PX7 *is expressed at very low levels in both strains. Hence it is possible that small differences in absolute transcript levels, which could be an outcome of genetic drift, may have resulted in the strong differentiation in expression of this gene between the strains.

### Gene expression associated with sex

In two strains of *A. gambiae *from East Africa, RSP and Kisumu, females were found to be intrinsically more tolerant to permethrin than males [24]. In the present study, the survival rates between the sexes in the resistant Odumasy strain differed only marginally. In order to assess whether this similarity in permethrin tolerance was correlated with expression of specific detoxification genes, labelled RNA from both sexes of the susceptible Kisumu strain was co-hybridised to the *detox chip*. Gene expression profiles between females and males of the pyrethroid resistant Odumasy strain were then indirectly estimated by comparing the data sets of all three microarray experiments, i.e. Odumasy females vs. Kisumu females, Kisumu females vs. Kisumu males, and Kisumu males vs. Odumasy males.

**Figure 2:**
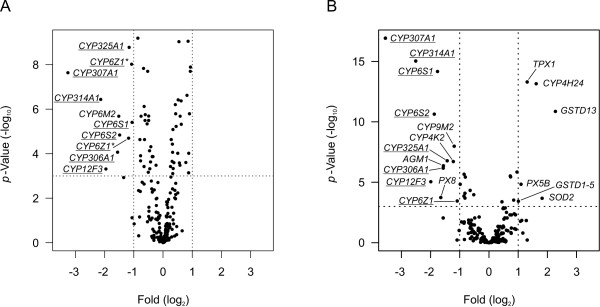
**Microarray analysis comparing females with males within each strain**. Data are shown for the gene expression levels of susceptible Kisumu females vs. males (A) and the permethrin resistant Odumasy females vs. males (B). Each dot represents the mean estimates, fold (*M*) and *p*-value (adjusted for multiple testing), for one gene from the complete microarray experiment. Names are shown for genes that are at least two-fold differentially expressed. Underlined names show genes that are common to both the Kisumu female-male and the Odumasy female-male comparison. Horizontal line represents the level of significance α = 0.001, and vertical lines indicate two-fold change threshold. *CYP6Z1 was spotted on the *detox chip *as both cDNA and 70-mer oligonucleotide probe [16].

Out of 233 gene probes on the microarray 61 genes were differentially expressed between the sexes in the Kisumu strain and 31 in the Odumasy strain (see Additional file 1). Among these genes ten showed a difference over two-fold in the Kisumu strain and 18 in the Odumasy strain (Figure 2 and Table 2). If one or more of the three enzymes, CYP6Z2, CYP6M2 and SOD3B, played a major role in permethrin resistance we would expect similar levels of expression in both sexes of the resistant strain, given that the resistance phenotype between females and males differed only slightly. None of these candidate genes was differentially expressed between females and males of the resistant Odumasy strain (see Additional file 1).

Eight genes found to be over-expressed by two-fold are common to both strains only in males (Figure 2 and Table 2). Of these eight genes the P450s, *CYP307A1 *and *CYP314A1*, show particularly high male biased expression. Orthologs of these genes along with *CYP306A1 *have been implicated in ecdysteroid metabolism in other insects [25-27]. The expression of genes involved in ecdysone and 20-hydroxyecdysone synthesis is tightly regulated and it is unlikely that these P450s play a major role in xenobiotic detoxification. In contrary, the five additional genes *CYP6S1*, *CYP6S2*, *CYP6Z1*, *CYP12F3 *and *CYP325A1 *belong to P450 families in which individual genes are associated with permethrin resistance ([16,19] and this study).

**Figure 3:**
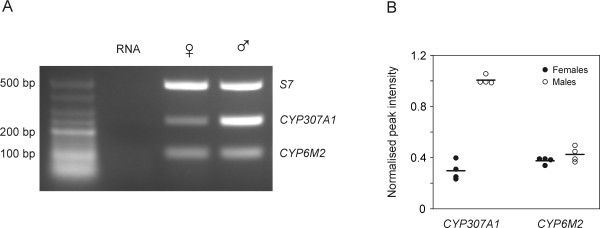
**Semi-quantitative RT-PCR validation of selected microarray data**. Gene expression levels of *CYP307A1 *and *CYP6M2 *between females and males of the susceptible Kisumu strain were compared. (A) Peak intensities of amplified cDNA products of the two target mRNAs were measured and normalised by the internal control, the ribosomal *S7 *product. Primers used to amplify cDNA are shown in Table 1. (B) Measurements were taken from four biological replicates for each sex (15 one-day adult mosquitoes per replicates). Dots represent normalised peak intensities for each replicate. Horizontal bars show mean values. *CYP307A1 *shows a 3.4-fold over-expression in males (one-sided *t*-test, *p *< 0.001) whereas *CYP6M2 *was 1.1-fold over-expressed in males but statistically not significant (one-sided *t*-test, *p *= 0.3).

The over-expression of *CYP307A1 *in males is confirmed by semi-quantitative RT-PCR (Figure 3). Nikou *et al*. [19], using quantitative RT-PCR, found *CYP6Z1 *also being over-expressed in males over females in the susceptible Kisumu strain in accordance with the microarray results in this study. Over-expression of *CYP6M2 *in Kisumu males over females was slightly higher in the semi-quantitative RT-PCR but was statistically not significant (Figure 3). However, the semi-quantitative RT-PCR is limited to the detection of very large fold changes and may not be sensitive enough to detect the lower fold change of *CYP6M2*. We are confident in our microarray results as the quantitative RT-PCR [19] and the overall pattern of the semi-quantitative RT-PCR are consistent with the microarray measurements.

## Discussion

Monitoring of metabolic resistance to insecticides in field populations currently relies on biochemical assays. These assays measure the correlation between increased enzymatic activities towards model substrates in resistant mosquitoes and are a general indicator of the enzyme families putatively involved. However, the sensitivity of these assays is questionable. For example, recently Lumjuan *et al*. [28] have shown that the GST responsible for DDT resistance, GSTE2, has very low levels of activity against the model substrate CDNB used to detected GST-based resistance mechanisms. Synergists, such as piperonyl butoxide can also be used to indicate the enzyme class involved although these require large numbers of live insects and hence are not practical for routine monitoring of resistance in field populations. Molecular tools such as the allele specific PCR [8,9] or the HOLA [29] assay developed to detect *kdr *mutations enable the frequency of resistance alleles to be measured directly. We still lack such tools to monitor metabolic resistance. Yet, this information is extremely valuable to mosquito control managers as it enables resistance to be detected when it first emerges in a population and before it may have a detectable effect on the resistance phenotype. However, the development of such molecular assays requires a detailed understanding of the mechanisms conferring resistance.

In the present study we identified six genes whose expression levels are associated with pyrethroid resistance in a recently colonised strain of *A. gambiae *from West Africa. Three of these six genes were over-expressed in both sexes of the resistant strain; two cytochrome P450s, *CYP6Z2 *and *CYP6M2*, and a member of the superoxide dismutase gene family, *SOD3B*. In females of the resistant strain the carboxylesterase *COEJHE5E *and a sigma class GST, *GSTS1-2*, were also found to be over-expressed while another P450, *CYP6Z3*, was over-expressed in the resistant males only.

Cytochrome P450-dependent monooxygenases are an important metabolic mechanism by which several insect species become resistant to insecticides [30]. Biochemical characterisation implicate P450s conferring pyrethroid resistance in the resistant East African *A. gambiae *RSP strain [31]. In *A. gambiae *P450 loci associated with pyrethroid resistance are mapped within a cluster in chromosome arm 3R [24]. This cluster also encompasses the three P450s, *CYP6Z2, CYP6Z3 *and *CYP6M2*, identified in this study among a subfamily of 14 *CYP6 *P450s. As survival rates after exposure to permethrin between females and males of the resistant strain vary only marginally, genes that are over-expressed in both sexes are more likely to be involved in conferring metabolic resistance. Arguably *CYP6Z2 *and *CYP6M2 *showing the highest transcript levels in both sexes are good candidate genes involved in metabolic detoxification of permethrin.

The CYP6 class of P450s is a rapidly evolving gene family [10] and there are no recognised orthologs for *CYP6Z2 *and *CYP6M2 *outside of the mosquito taxa. The most similar P450s to CYP6Z2 as retrieved by BLAST [32] found in other insect species are CYP6D3 in *Musca domestica *(44% amino sequence identity) and CYP6D4 in *Drosophila melanogaster *(43% amino sequence identity). Intriguingly *CYP6D3 *is over-expressed in several housefly strains resistant to permethrin [33] while deletion of *CYP6D4 *in *D. melanogaster *did not alter the toxicity of several insecticides including pyrethroids [34]. P450s most similar to CYP6M2 identified using BLAST [32] in *A. gambiae *are the CYP6N3 in *Aedes albopictus *(54% amino sequence identity), CYP6BB1 in the eastern saltmarsh mosquito, *Ochlerotatus sollicitans*, (49% amino sequence identity), and CYP6A5 in *M. domestica *(49% amino sequence identity). In *D. melanogaster *the protein showing the highest similarity with CYP6M2 is CYP6A23 (48% amino sequence identity). To our knowledge these putatively related genes have yet not been associated with pyrethroid resistance. Enzymes of the CYP6 family are, however, generally involved in detoxification of xenobiotics in insects [30]. Functional characterisation of these enzymes is required to demonstrate the biological role of *CYP6Z2 *and *CYP6M2 *in pyrethroid resistance in *A. gambiae*.

Along with the neurotoxic effect, pyrethroids may induce oxidative stress which increases the toxicity of the insecticide [15]. Elevated levels of the SOD3B might increase pyrethroid resistance as superoxide dismutase enzymes show antioxidant properties protecting organisms from the damaging effects of reactive oxygen species [35]. While *SOD3B *was up-regulated in the Odumasy strain superoxide dismutases were not found to be differentially expressed in the permethrin resistant RSP strain [16,36]. Among the three candidate genes apparent in both sexes of the resistant strain, *SOD3B *shows the lowest expression levels and higher variability – indicated by its higher *p*-value – across measurements, assigning this gene a lower rank. Likewise, *GSTS1-2 *– over-expressed in females of the resistant strain – although unlikely to be directly involved in pyrethroid metabolism may confer increased pyrethroid tolerance as a result of its antioxidant properties. In *D. melanogaster *a sigma class GST, *DmGSTS1*, is thought to have a function in protection against oxidative injury [37] although it was initially subscribed a structural function in the indirect flight muscle [38]. Females of the permethrin resistant Odumasy strain also show higher transcript levels for COEJHE5E which is similar to the *D. melanogaster *gene product drome-CG8425. drome-CG8425 has been identified as a juvenile hormone esterase [39]. To our knowledge there is no implication that this gene is involved in pyrethroid detoxification and may rather reflect strain specific differences in the metabolism of the female reproductive system.

The comparison between females and males in each strain revealed a larger set of genes being differentially expressed than the strain comparison within each sex. This is not surprising as many traits such as reproduction, host seeking, blood feeding etc. differ between the sexes. In *A. gambiae*, whole genome arrays show sex-biased gene expression pattern [40], and a comprehensive microarray experiment looking at the contribution of sex, genotype and age to transcriptional variance in *D. melanogaster *found that most of the variation is attributed to sex [41]. While no gene has been found to be consistently above two-fold over-expressed in females among both the susceptible and resistant strain, eight P450 genes were over-expressed in males over females in both strains. Five P450s, *CYP6S1*, *CYP6S2*, *CYP6Z1*, *CYP12F3 *and *CYP325A1*, belong to families implicated in xenobiotic detoxification. The CYP6 members are implicated in pyrethroid resistance, *CYP6M2 *(this study) and *CYP6Z1 *[16,19], while expression of *CYP325A1 *and *CYP12F3 *have not been correlated with insecticide resistance. However, expression of another member of the *CYP325 *family, *CYP325A3*, has been associated with insecticide resistance in the pyrethroid resistant RSP strain [16]. Putatively all five P450s may play a role in xenobiotic detoxification. Orthologs of the other three over-expressed P450s in males, *CYP307A1*, *CYP314A1*, and *CYP306A1*, are all involved in the ecdysteroid hormone biosynthesis in *D. melanogaster *[25-27]. Ecdysteroid hormones control a range of biological functions including molting, behaviour, the development of the nervous system, reproduction, and production of sex pheromones [42]. It is possible that the biosynthesis of ecdysteroid hormones such as those triggering spermatogenesis are increased in males over females during the early stages in adult anopheline mosquitoes.

Field studies in western Kenya revealed the presence of target site insensitivity and metabolic resistance in the same *A. gambiae *population where pyrethroid-treated bed nets are being widely used [6,9,31]. Measurements of transcript levels in the permethrin resistant RSP strain originating from the same region showed elevated levels of *CYP6Z1 *and *GSTE2 *[16,19]. In the West African Odumasy strain neither of these genes is associated with permethrin resistance. In contrast *GSTE2 *levels are depressed in males from the resistant versus the susceptible strain. Here, two alternative P450s, *CYP6Z2 *and *CYP6M2*, are highly over-expressed in females and males.

The Odumasy strain also shows high frequency (84%) of the West African type *kdr *allele in field populations [43]. This *kdr *allele differs from the East African type by a different mutation at the same amino acid position conferring permethrin resistance [8,9]. The present data implies that, as for target site insensitivity, metabolic resistance to the same insecticide class may be due to alternative mechanisms. In contrast to target site insensitivity – where only a few set of alternative alleles are possible as most alleles would alter the protein's overall structure – extensive gene duplication within the mosquito detoxification gene family may enable up-regulation of various enzymes that alternatively contribute to insecticide resistance. Analyses of expression patterns in a series of susceptible and resistant *D. melanogaster *strains revealed that P450-mediated metabolic resistance evolves via the differential selection of multiple P450s through selection to the same insecticides [44]. Similarly in house flies, Scott and Kasai [45] found different P450s being selected against permethrin in different populations. One of the factors causing evolutionary plasticity to the same insecticide could be differential selection pressures as a function of actual insecticide dosages encountered by mosquitoes in the field. It has been suggested if selection takes repeatedly place within the normal response of an insect population such doses of insecticide will select for polygenic resistance while those outside the normal response will select for monogenic resistance [46]. Selection pressures may be higher where insecticides are widely used in agriculture as opposed to pyrethroid-treated bed nets. While the intensity of application certainly plays an important role, temporal fluctuations in insecticide dosages may be equally important. For example, Diabate *et al*. [47] found seasonal variation of insecticide resistance in *A. gambiae *field populations in relation to the use of insecticides in agriculture. Given the overall heterogeneity in insecticide coverage it may well be that in some circumstances selection will favour either mono- or polygenic resistance.

Barriers in gene flow such as the Rift Valley between East and West Kenyan populations or geographical distance between populations may also favour multiple origins of insecticide resistance. Such barriers have been suggested to explain variation in the genetic diversity among African *A. gambiae *populations [20]. Noteworthy, the East African *kdr *allele type reported from the permethrin resistant *A. gambiae *in western Kenya is not found in coastal Kenya [6]. Likewise some of the differences in gene expression patterns found in this study may be attributable to geographic variation rather than the observed phenotype. Nevertheless, both loci, *CYP6Z2 and CYP6M2*, identified in this study are mapped within a cluster of P450s on chromosome 3R previously associated with pyrethroid resistance [24].

An unresolved issue is how much of the observed resistance phenotype is attributable to *kdr*. Target site and metabolic mechanisms may act synergistically [9] and the detection of both mechanisms is therefore crucial. The contribution of the two mechanisms in the Odumasy strain and other populations needs further research. Selection experiments on field collected mosquitoes where both *kdr *frequencies and gene expression levels are measured would provide the most complete evidence. Additionally, studies comparing selected and parental strains from various field sites across Africa are needed to shed more light on the origin and evolution of metabolic resistance in field populations of *A. gambiae*.

## Conclusion

To date pyrethroids are the only highly effective class of commercially insecticides available to treat bednets. The present data suggest that there may be multiple metabolic resistance mechanisms found in *A. gambiae *populations which pose a severe challenge to the sustainability of malaria vector control programmes. New insecticides and a comprehensive screening tool for the detection and management of insecticide resistance are urgently required for use in malaria epidemic regions.

## Methods

### Mosquito strains and selection to insecticide

Two *A. gambiae *s.s. strains were compared; the permethrin resistant Odumasy strain vs. the insecticide susceptible Kisumu strain. Both strains belong to the molecular "S" form. The Odumasy strain originated from Odumasy, Southern Ghana, and was colonised in the laboratory from field collected specimens in 2002. The Odumasy strain shows high frequency (84%) of the West African type *kdr *allele in field populations [43]. In our lab colony the *kdr *allele is fixed (Amy Lynd and Nadine Randle, unpublished data). The field collected Odumasy strain has been repeatedly selected in the laboratory by exposure to filter paper impregnated with 0.75% permethrin according to WHO standard procedures [48]. Both sexes showed low mortality after a one-hour exposure (females: 26%, *N *= 224; males: 38%, *N *= 297) with females being slightly more resistant (χ^2^-test, *p *= 0.0045). To our knowledge this is the first resistant strain of *A. gambiae *s.s. "S" form originating from West Africa which has been successfully reared under laboratory conditions. The Kisumu strain originated from Kisumu, Western Kenya, and is susceptible to permethrin [3]. The susceptibility of the Kisumu lab strain has been repeatedly demonstrated using the same WHO protocol. No survivors were recorded in the Kisumu strain at this discriminatory dose. Both strains were reared under constant laboratory conditions (26°, 80% RH) and a 12:12 hours light:dark cycle. Larvae were fed with finely grounded fish food (TetraMin) *ad libitum*. Adult mosquitoes were provided with water and sugar and fed with human blood twice a week.

### Target preparation and microarray hybridisations

Adult mosquitoes were collected the morning after emergence and frozen at -80°C. Total RNA was extracted using the RiboPure Kit (Ambion) from batches of 10 to 15 mosquitoes. A batch contained mosquitoes from the same generation collected on the same day. After extraction, RNA quantity was measured using a Nanodrop spectrophotometer (Nanodrop Technologies, Oxfordshire, UK). 8 μg of total RNA from each batch were first reverse transcribed to single-stranded cDNA using an oligo d(T)_24 _primer (Sigma) and Superscript III reverse transcriptase (Invitrogen). Single-stranded cDNA was then converted into double-stranded cDNA. The double-stranded cDNA was used as a template to generate single-stranded antisense RNA (aRNA) in one amplification round using the MEGAscript T7 Kit (Ambion). Amplified aRNA was purified using the RNeasy Mini Kit (Qiagen). Prior to fluorescent labelling aRNA quantity and quality were assessed by a spectrophotometer (Nanodrop) and agarose gel electrophoresis. Final target samples were prepared from 8 μg of sample aRNA and 4 μg RNA control spike mix (Lucidea Universal Score Card, Amersham Biosciences). Fluorescent nucleotides, Cyanine 3-dUTP (Cy3) or Cyanine 5-dUTP (Cy5) (GeneBeam, Enzo) respectively, were incorporated into first-strand cDNA by reverse transcriptase using random hexamer primers (Life Technologies) and Superscript III reverse transcriptase (Invitrogen). After degradation of the aRNA template strand using 1 μl 1 M NaOH, 20 mM EDTA and incubating for 5 min at 70°C, cDNA targets were pooled together and purified using the CyScribe GFX Purification Kit (Amersham Biosciences). Dye incorporation efficiency was measured using a spectrophotometer (Nanodrop) and 5 μg of poly dA oligo (Amersham Biosciences) was added to prevent non-specific hybridisation. The labelled cDNA targets were vacuum dried and re-suspended in 15 μl formamide-based hybridisation buffer (Corning). Labelled targets were hybridised to the *detox chip *[16] for 16 h at 42°C following the preparation of the glass slides using the Universal Hybridisation Kit (Corning) according to the manufacturer's recommendations. The *detox chip *is a custom-made microarray chip containing probes from 233 *A. gambiae *genes associated with metabolic resistance; 103 cytochrome P450 monooxygenases (P450s), 31 carboxylesterases (COEs), 35 glutathione-S-transferases (GSTs), 41 redox genes, 5 ATP-binding-cassette transporters, and various control genes [16]. After incubation slides were washed according to manufacturer's instructions and intensity images were scanned by a Genepix Personal 4100a microarray scanner (Axon instruments) at a resolution of 10 μm.

### Microarray experimental design

In a first set of microarray experiments transcription levels in resistant mosquitoes were compared to levels in susceptible mosquitoes. The experiments were carried out separately for females and males. In addition to the strain comparison transcript levels were then compared between the sexes in each strain. Females and males of the Kisumu strain were compared directly in an additional microarray experiment while transcript levels between females and males of the Odumasy strain were estimated indirectly by combining all above comparisons (see below). The Odumasy colony was difficult to maintain at a sustainable level to collect sufficient material for all comparisons.

Each set of microarray experiments comparing the two strains consisted of six hybridisations comprising three biological and two technical replicates. Each biological replicate was split into two aliquots which were co-hybridised to two arrays while the Cy3 and Cy5 labels were swapped between hybridisations. For the sex comparison within the susceptible Kisumu strain two independent biological replicates with two technical repeats were performed.

### Microarray data analysis

After visual inspection spot and background intensities were calculated from the scanned array images using GenePix Pro 5.1 software (Axon Instruments). Subsequent data analysis was performed using the Limma 2.4.1 software package [49] for R 2.3.0, available from the CRAN repository [50]. Limma is part of the Bioconductor project [51]. For each spot background intensities were subtracted from the total spot intensities using the method *normexp *and an offset of 50 as recommended for GenePix data by the Limma user's guide. The *detox chip *contains 40 calibration spots that, when hybridised with labelled samples containing the mRNA spike mix, result in 1:1 ratios representing a concentration range from 1 pg to 30 ng per 2 μl of mRNA spike mix. Intensity values from the calibration spots were used in addition to the gene spot intensities for data normalisation. In a first step, background corrected intensities from the red, *R *(Cy5), and the green, *G *(Cy3), channel were transformed to intensity log-ratios, *M *= log_2 _*R*/*G*, and their corresponding geometrical means, *A *= (log_2 _*R *+ log_2 _*G*)/2. Then within each array *M*-values were normalised as a function of *A *using the *loess *scatter plot smoothing function. In a second normalisation step the *M*-values from each array were scaled to equalise the median absolute value across all arrays to account for technical biases between replicate hybridisations [52]. The between arrays normalisation was first performed for each experiment separately taking only those slides into account that were used in each experiment. For the comparison between Odumasy females and males where expression ratios were estimated indirectly by using the information from all experiments the within arrays normalisation was repeated across all arrays included in the analysis.

The association of gene expression with the phenotypic variable, i.e. strain or sex, was assessed by fitting linear models to estimate the gene expression for each gene on the array. To account for non-independent measurements between technical replicates estimates were first obtained from each biological replicate and then averaged between the biological replicates. Contrasts, linear combinations of the coefficients, were then tested for significance. For the comparison between Odumasy females and males the following linear combination was used: *M*_1 _- *M*_2 _+ *M*_3 _with *M*_1 _= log_2_(*Odumasy females vs. Kisumu females*), *M*_2 _= log_2_(*Odumasy males vs. Kisumu males*) and *M*_3 _= log_2_(*Kisumu females vs. Kisumu males*). As each spot is replicated four times on the *detox chip*, valuable information was available on the gene-wise within-array variability which was used to estimate the technical, spatial variation on the array. The correlation between the four spots was taken into account by fitting separate linear models to the expression data for each gene but with a common value for the between-replicate correlation [53]. Compared with simply averaging replicate spots, this method greatly improves the precision with which the gene wise variances are estimated. The significance was assessed with a moderated *t*-statistic which shows a robust behaviour for small numbers of arrays and allows for incomplete data from missing spots [54]. To account for multiple testing *p*-values were adjusted adopting the approach of Benjamini and Hochberg [55] to control for the false discovery rate as described in [49]. In order to define a set of candidate genes only those genes were considered which showed at least a two-fold change in expression, i.e. *M *= 1, and an adjusted *p*-value less than *q *= 0.001.

As a control a calibration experiment was performed in which two aliquots derived from the same sample were co-hybridised to two arrays with reverse-labelling. As expected, none of the gene probes came out significantly in the calibration experiment supporting the statistical approach chosen above (data not shown). In addition to the control experiment a semi-quantitative RT-PCR was performed to measure expression levels of two candidate genes (see below).

### Semi-quantitative RT-PCR

To validate microarray data semi-quantitative RT-PCR was performed. RNA was first extracted from four replicate sets (15 mosquitoes per replicate) of one-day old adult males or females of the Kisumu strain. Total RNA was extracted using the methods described above followed by DNase treatment (RiboPure Kit, Ambion) to eliminate any genomic DNA. Extracted total RNA was reverse transcribed to cDNA using an oligo d(T)_14 _primer and Superscript III reverse transcriptase (Promega). PCR primers (Table 3) were designed to amplify two P450 genes, *CYP307A1 *and *CYP6M2*, along with the ribosomal gene *S7 *[GenBank: AY380336] [56] which served as an internal standard to account for differences in initial cDNA and reaction efficiency. 0.5 μl cDNA was used as templates in the PCR reactions which contained 7.5 μl 2× Multiplex PCR Master Mix (Qiagen), 0.2 μM of each forward and reverse primer, *CYP307A1 *and *CYP6M2*, and 0.05 μM of each forward and reverse primer for *S7*. PCR reactions were carried out in a MJ Research PTC thermal cycler. An initial activation step of 15 minutes at 95°C was followed by 20 seconds at 94°C, 20 seconds at 55°C and 30 seconds at 72°C for 33 cycles. The conditions were chosen so that all of the cDNAs amplified were still within the dynamic range to avoid saturation of one of the templates at the end of the PCR protocol. To rule out genomic DNA contamination RNA was used as negative control. The PCR products were loaded onto ethidium bromide-stained 2% agarose gels in TAE buffer. Images of the gels were acquired with GeneSnap 4 (Syngene) and raw peak intensities analysed using GeneTools 3 (Syngene). Peak intensities of the *CYP307A1 *and *CYP6M2 *bands were normalised by the peak intensity of *S7*.

## Authors' contributions

PM conceived and carried out the microarray experiments, performed the statistical analysis and drafted the manuscript. MJD and HR participated in the study design and helped to draft the manuscript. All authors read and approved the final manuscript.

## Supplementary Material

Additional file 1 **Odumasy expression data ALL**. This table contains a complete list of the current microarray analysis including fold expression and *p*-values for all genes represented on the *detox chip*. Click here for file

## Figures and Tables

**Table 1:** Differential gene expression between the permethrin resistant Odumasy and the susceptible Kisumu strain

Gene	Function	Cytological position	Accession number	Females		Males	
				
				Fold	*p*-Value	Fold	*p*-Value
Genes over-expressed in resistant Odumasy strain							

*CYP6M2*	Cytochrome P450 monooxygenase	3R	[GenBank: AY193729]	5.2	7.94 × 10^-05^	3.2	1.29 × 10^-11^
*CYP6Z2*	Cytochrome P450 monooxygenase	3R	[GenBank: AF487780]	4.2	8.62 × 10^-07^	8.0	2.27 × 10^-11^
*SOD3B*	Cu-Zn superoxide dismutase	3L	[GenBank: AY745234, TIGR: TC54196]	3.5	8.11 × 10^-04^	2.0	1.00 × 10^-04^
*GSTS1-2*	Glutathione S-transferase	3L	GenBank: AF513639]	2.5	2.91 × 10^-04^	-	n.s.
*COEJHE5E*	Esterase	2L	[TIGR: TC77639]	2.0	3.86 × 10^-09^	1.8	1.67 × 10^-08^
*CYP6Z3*	Cytochrome P450 monooxygenase	3R	[GenBank: AY193727]	-	n.s.	3.0	1.54 × 10^-^^07^

Genes over-expressed in the susceptible Kisumu strain							

*GSTE2*	Glutathione S-transferase	3R	[GenBank: AF316636]	-	n.s.	2.7	4.61 × 10^-04^
*CYP6P1*	Cytochrome P450 monooxygenase	2R	[GenBank: AY028785]	2.3	1.06 × 10^-06^	1.6	7.32 × 10^-11^
*CYP6AJ1*	Cytochrome P450 monooxygenase	3L	[GenBank: AY745226]	2.0	8.76 × 10^-06^	-	n.s.
*PX7*	Peroxidase	3L	[GenBank: AY752901]	-	n.s.	7.5	6.45 × 10^-06^

Only genes are listed which showed at least a two-fold difference in one or both sexes. *p*-values were corrected for multiple testing as described in the materials and methods section. n.s., *p *≥ 0.001.

**Table 2:** Differential gene expression between females and males within each strain

Gene	Function	Cytological position	Accession number	Kisumu strain		Odumasy strain	
				
				Fold	*p*-Value	Fold	*p*-Value
Genes over-expressed in males							

*CYP307A1*	Cytochrome P450 monooxygenase	X	[GenBank: AY745209]	9.6	2.31 × 10^-08^	11.7	1.20 × 10^-17^
*CYP314A1*	Cytochrome P450 monooxygenase	2R	[GenBank: AY745210]	4.4	3.67 × 10^-07^	5.7	9.29 × 10^-16^
*CYP12F3*	Cytochrome P450 monooxygenase	3R	[GenBank: AY176049]	3.9	4.88 × 10^-04^	4.0	9.04 × 10^-06^
*CYP306A1*	Cytochrome P450 monooxygenase	2R	[GenBank: AB162964]	2.9	8.59 × 10^-05^	3.0	6.76 × 10^-07^
*CYP6M2*	Cytochrome P450 monooxygenase	3R	[GenBank: AY193729]	2.9	2.08 × 10^-06^	-	n.s.
*CYP6S2*	Cytochrome P450 monooxygenase	3R	[GenBank: AY062207]	2.8	1.45 × 10^-05^	3.7	2.31 × 10^-11^
CYP6Z1*	Cytochrome P450 monooxygenase	3R	[GenBank: AF487535]	2.3	2.01 × 10^-05^	-	n.s.
*CYP325A1*	Cytochrome P450 monooxygenase	2R	[GenBank: AY748842]	2.2	1.68 × 10^-09^	2.7	1.66 × 10^-07^
CYP6Z1*	Cytochrome P450 monooxygenase	3R	[GenBank: AF487535]	2.1	9.58 × 10^-09^	2.1	3.55 × 10^-04^
*CYP6S1*	Cytochrome P450 monooxygenase	3R	[GenBank: AY028784]	2.1	3.92 × 10^-06^	3.4	6.83 × 10^-15^
*AGM1*	Midgut maltase-like protein	3L	[GenBank: X87410]	1.6	7.58 × 10^-07^	3.0	4.33 × 10^-07^
*CYP4K2*	Cytochrome P450 monooxygenase	2R	[GenBank: AY748834]	1.5	4.59 × 10^-06^	2.3	1.93 × 10^-07^
*PX8*	Peroxidase	3L	[GenBank: AY752902]	-	n.s.	3.1	1.83 × 10^-04^
*CYP9M2*	Cytochrome P450 monooxygenase	3R	[GenBank: AY748851]	-	n.s.	2.3	1.04 × 10^-08^

Genes over-expressed in females							

*GSTD13*	Glutathione S-transferase	X	[GenBank: AF515521]	1.9	1.99 × 10^-08^	4.8	1.38 × 10^-11^
*PX5B*	Peroxidase	3L	[GenBank: AY752899]	1.8	7.25 × 10^-04^	2.1	1.47 × 10^-05^
*TPX1*	Thioredoxin peroxidase	X	[GenBank: AY745234, TIGR: TC49541]	1.8	2.39 × 10^-07^	2.5	5.02 × 10^-14^
*SOD2*	Cu-Zn superoxide dismutase	2L	[GenBank: AY524130, TIGR: TC39286]	1.5	4.25 × 10^-07^	3.5	2.11 × 10^-04^
*CYP4H24*	Cytochrome P450 monooxygenase	X	[GenBank: AY062206]	-	n.s.	3.1	7.08 × 10^-14^
*GSTD1-5*	Glutathione S-transferase	2R	[GenBank: Z81291]	-	n.s.	2.0	3.72 × 10^-04^

Only genes are listed which showed at least a two-fold difference in one or both strains. *p*-values were corrected for multiple testing as described in the materials and methods section. n.s., *p *≥ 0.001. *CYP6Z1 was spotted on the *detox chip *as both cDNA and 70-mer oligonucleotide probe [16].

**Table 3:** Sequences of oligonucleotide primers used in semi-quantitative RT-PCR validation experiments

Gene	Accession number	Primer	Sequence (5' to 3')	Transcript length
*CYP6M2*	[GenBank: AY193729]	CYP6M2_F CYP6M2_R	GTATGATGCAGGCCCGTATAG GCCATAATGAAACTCTCCTTCG	112 bp
*CYP307A1*	[GenBank: AY745209]	CYP307A1_F CYP307A1_R	ACTACGAGCTGAACACGAGTGA ATGCAGGTCCGTTTACCAATAC	253 bp
*S7 ribosomal*	[GenBank: AY380336]	SPC SPD	GTGCCGGTGCCGAAACAGAA AGCACAAACACTCCAATAATCAAG	472 bp

Only genes are listed which showed at least a two-fold difference in one or both strains. *p*-values were corrected for multiple testing as described in the materials and methods section. n.s., *p *= 0.001. *CYP6Z1 was spotted on the *detox chip *as both cDNA and 70-mer oligonucleotide probe [16].
